# Reduced Glomerular Filtration Rate and Its Association with Clinical Outcome in Older Patients at Risk of Vascular Events: Secondary Analysis

**DOI:** 10.1371/journal.pmed.1000016

**Published:** 2009-01-20

**Authors:** Ian Ford, Vladimir Bezlyak, David J Stott, Naveed Sattar, Chris J Packard, Ivan Perry, Brendan M Buckley, J. Wouter Jukema, Anton J. M de Craen, Rudi G. J Westendorp, James Shepherd

**Affiliations:** 1 Robertson Centre for Biostatistics, University of Glasgow, Glasgow, Scotland; 2 Academic Section of Geriatric Medicine, Cardiovascular Division, University of Glasgow, Glasgow, Scotland; 3 Department of Vascular Biochemistry, North Glasgow Division, Greater Glasgow Health Board, Glasgow, Scotland; 4 Department of Pharmacology and Therapeutics, Cork University Hospital, Wilton, Cork, Ireland; 5 Department of Cardiology, Leiden University Medical Centre, Leiden, The Netherlands; 6 Section of Gerontology and Geriatrics, Leiden University Medical Centre, Leiden, The Netherlands; Istituto Mario Negri, Italy

## Abstract

**Background:**

Reduced glomerular filtration rate (GFR) is associated with increased cardiovascular risk in young and middle aged individuals. Associations with cardiovascular disease and mortality in older people are less clearly established. We aimed to determine the predictive value of the GFR for mortality and morbidity using data from the 5,804 participants randomized in the Prospective Study of Pravastatin in the Elderly at Risk (PROSPER).

**Methods and Findings:**

Glomerular filtration rate was estimated (eGFR) using the Modification of Diet in Renal Disease equation and was categorized in the ranges ([20–40], [40–50], [50–60]) ≥ 60 ml/min/1.73 m^2^. Baseline risk factors were analysed by category of eGFR, with and without adjustment for other risk factors. The associations between baseline eGFR and morbidity and mortality outcomes, accrued after an average of 3.2 y, were investigated using Cox proportional hazard models adjusting for traditional risk factors. We tested for evidence of an interaction between the benefit of statin treatment and baseline eGFR status. Age, low-density lipoprotein (LDL) and high-density lipoprotein (HDL) cholesterol, C-reactive protein (CRP), body mass index, fasting glucose, female sex, histories of hypertension and vascular disease were associated with eGFR (*p =* 0.001 or less) after adjustment for other risk factors. Low eGFR was independently associated with risk of all cause mortality, vascular mortality, and other noncancer mortality and with fatal and nonfatal coronary and heart failure events (hazard ratios adjusted for CRP and other risk factors (95% confidence intervals [CIs]) for eGFR < 40 ml/min/1.73m^2^ relative to eGFR ≥ 60 ml/min/1.73m^2^ respectively 2.04 (1.48–2.80), 2.37 (1.53–3.67), 3.52 (1.78–6.96), 1.64 (1.18–2.27), 3.31 (2.03–5.41). There were no nominally statistically significant interactions (*p* < 0.05) between randomized treatment allocation and eGFR for clinical outcomes, with the exception of the outcome of coronary heart disease death or nonfatal myocardial infarction (*p =* 0.021), with the interaction suggesting increased benefit of statin treatment in subjects with impaired GFRs.

**Conclusions:**

We have established that, in an elderly population over the age of 70 y, impaired GFR is associated with female sex, with presence of vascular disease, and with levels of other risk factors that would be associated with increased risk of vascular disease. Further, impaired GFR is independently associated with significant levels of increased risk of all cause mortality and fatal vascular events and with composite fatal and nonfatal coronary and heart failure outcomes. Our analyses of the benefits of statin treatment in relation to baseline GFR suggest that there is no reason to exclude elderly patients with impaired renal function from treatment with a statin.

## Abstract 2

Using data from the PROSPER trial, Ian Ford and colleagues investigate whether reduced glomerular filtration rate is associated with cardiovascular and mortality risk among elderly people.

## Editors' Summary

**Background.:**

Cardiovascular disease (CVD)—disease that affects the heart and/or the blood vessels—is a common cause of death in developed countries. In the USA, for example, the single leading cause of death is coronary heart disease, a CVD in which narrowing of the heart's blood vessels slows or stops the blood supply to the heart and eventually causes a heart attack. Other types of CVD include stroke (in which narrowing of the blood vessels interrupts the brain's blood supply) and heart failure (a condition in which the heart can no longer pump enough blood to the rest of the body). Many factors increase the risk of developing CVD, including high blood pressure (hypertension), high blood cholesterol, having diabetes, smoking, and being overweight. Tools such as the “Framingham risk calculator” assess an individual's overall CVD risk by taking these and other risk factors into account. CVD risk can be minimized by taking drugs to reduce blood pressure or cholesterol levels (for example, pravastatin) and by making lifestyle changes.

**Why Was This Study Done?:**

Another potential risk factor for CVD is impaired kidney (renal) function. In healthy people, the kidneys filter waste products and excess fluid out of the blood. A reduced “estimated glomerular filtration rate” (eGFR), which indicates impaired renal function, is associated with increased CVD in young and middle-aged people and increased all-cause and cardiovascular death in people who have vascular disease. But is reduced eGFR also associated with CVD and death in older people? If it is, it would be worth encouraging elderly people with reduced eGFR to avoid other CVD risk factors. In this study, the researchers determine the predictive value of eGFR for all-cause and vascular mortality (deaths caused by CVD) and for incident vascular events (a first heart attack, stroke, or heart failure) using data from the Prospective Study of Pravastatin in the Elderly at Risk (PROSPER). This clinical trial examined pravastatin's effects on CVD development among 70–82 year olds with pre-existing vascular disease or an increased risk of CVD because of smoking, hypertension, or diabetes.

**What Did the Researchers Do and Find?:**

The trial participants were divided into four groups based on their eGFR at the start of the study. The researchers then investigated the association between baseline CVD risk factors and baseline eGFR and between baseline eGFR and vascular events and deaths that occurred during the 3-year study. Several established CVD risk factors were associated with a reduced eGFR after allowing for other risk factors. In addition, people with a low eGFR (between 20 and 40 units) were twice as likely to die from any cause as people with an eGFR above 60 units (the normal eGFR for a young person is 100 units; eGFR decreases with age) and more than three times as likely to have nonfatal coronary heart disease or heart failure. A low eGFR also increased the risk of vascular mortality, other noncancer deaths, and fatal coronary heart disease and heart failure. Finally, pravastatin treatment reduced coronary heart disease deaths and nonfatal heart attacks most effectively among participants with the greatest degree of eGFR impairment.

**What Do These Findings Mean?:**

These findings suggest that, in elderly people, impaired renal function is associated with levels of established CVD risk factors that increase the risk of vascular disease. They also suggest that impaired kidney function increases the risk of all-cause mortality, fatal vascular events, and fatal and nonfatal coronary heat disease and heart failure. Because the study participants were carefully chosen for inclusion in PROSPER, these findings may not be generalizable to all elderly people with vascular disease or vascular disease risk factors. Nevertheless, increased efforts should probably be made to encourage elderly people with reduced eGFR and other vascular risk factors to make lifestyle changes to reduce their overall CVD risk. Finally, although the effect of statins in elderly patients with renal dysfunction needs to be examined further, these findings suggest that this group of patients should benefit at least as much from statins as elderly patients with healthy kidneys.

**Additional Information.:**

Please access these Web sites via the online version of this summary at http://dx.doi.org/10.1371/journal.pmed.1000016.

- The MedlinePlus Encyclopedia has pages on coronary heart disease, stroke, and heart failure (in English and Spanish)
- MedlinePlus provides links to many other sources of information on heart disease, vascular disease, and stroke (in English and Spanish)
- The US National Institute of Diabetes and Digestive and Kidney Diseases provides information on how the kidneys work and what can go wrong with them, including a list of links to further information about kidney disease
- The American Heart Association provides information on all aspects of cardiovascular disease for patients, caregivers, and professionals (in several languages)
- More information about PROSPER is available on the Web site of the Vascular Biochemistry Department of the University of Glasgow

## Introduction

Reduced glomerular filtration rate (GFR) is associated with increased cardiovascular risk in young and middle aged individuals [1]. It is predictive of increased all-cause and cardiovascular mortality in individuals with vascular disease [2], greater severity of vascular disease [3], and worse outcome including increased mortality in heart failure [4–6] and after acute myocardial infarction [7,8]. Tools designed to assess cardiovascular risk in middle-aged individuals, such as the Framingham risk calculator, consistently underestimate event rates in patients with reduced GFR [9].

The associations of reduced GFR with incident cardiovascular disease and mortality in older people are less clearly established. In healthy community-dwelling older people in the US Cardiovascular Health Study, raised serum creatinine and reduced estimated GFR had no significant association with cardiovascular events or total mortality [10]. In elderly men in the British Regional Heart Study, where outcome was restricted to mortality, the association between mild-to-moderate renal insufficiency and outcome was explained to a significant extent by other risk factors [11].

We aimed to determine the predictive value of the GFR for all-cause and vascular mortality and incident vascular events in older people at risk of vascular disease using data from the Prospective Study of Pravastatin in the Elderly at Risk (PROSPER). This was a clinical trial of the HMG co-enzyme A reductase inhibitor pravastatin versus placebo in elderly men and women with a history of, or at risk for, vascular disease [12–14].

## Methods

The PROSPER study included 5,804 men and women between the ages of 70 and 82 y, recruited in Scotland, Ireland, and The Netherlands. The design, baseline characteristics of the participants, and the primary study results have been published elsewhere [12–14]. Selection criteria included pre-existing vascular disease (coronary, cerebral, or peripheral) or increased risk of such disease because of smoking, hypertension, or diabetes. Fasting baseline plasma total cholesterol was in the range 4.0–9.0 mmol/l and triglyceride concentrations were less than 6.0 mmol/l. Individuals with baseline creatinine levels over 200 μmol/l were excluded. Ethics committees of all trial centres approved the protocol, and all participants provided written informed consent.

Participants were randomised to receive either pravastatin 40 mg or matching placebo, to be taken once daily. Participants attended trial visits every 3 mo, with a mean follow-up of 3.2 y. Lipoprotein profiles were measured at the Centre for Disease Control–certified central lipoprotein laboratory in Glasgow. Serum creatinine levels were measured at central laboratories, one in each of the three participating countries. GFR was estimated using the Modification of Diet in Renal Disease equation [15,16]:

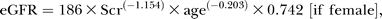
where Scr denotes serum creatinine level (mg/dl). It is assumed that all participants were of Northern European descent.

The primary outcome of the original trial was the combined endpoint of death from CHD, nonfatal myocardial infarction, and fatal or nonfatal stroke, assessed in the entire cohort. All deaths and all cancers were recorded. Tertiary endpoints included an assessment of transient ischemic attacks. All clinical endpoints were adjudicated by an expert study endpoints committee blinded to randomised study medication and using predefined criteria.

Outcomes studied in this report include death from all causes, and deaths from vascular causes, cancer and deaths from other noncancer noncardiovascular causes, the composite outcome of coronary heart disease death or nonfatal myocardial infarction, cerebrovascular events (fatal or nonfatal stroke or transient ischaemic attack), and the composite of death or hospitalization due to heart failure.

All study data were processed and analysed at the study Data Centre in The Robertson Centre for Biostatistics, University of Glasgow. Statistical analysis of baseline characteristics was based on a comparison among subgroups based on ranges of eGFR ([20–40], [40–50], [50–60] ≥ 60 ml/min/1.73 m^2^), first unadjusted and then adjusted for the potentially confounding effects of study inclusion criteria (histories of vascular disease, diabetes, hypertension, and current smoking status, all categorized as yes/no) as well as age and gender. Diabetes was defined to be self-reported history, a fasting blood glucose concentration of 7.0 mmol/l or greater, or a blood glucose measurement of 11.1 mmol/l or greater when fasting status was uncertain, or self-reported use of antidiabetic drugs (any oral hypoglycaemic agent or insulin). Only 18 participants had eGFR < 30 ml/min/1.73 m^2^, making impracticable further subdivision at the bottom end of the eGFR range. Continuous variables are summarized by means and standard deviations and compared by one-way analysis of variance or covariance as appropriate with the calculation of a *p*-value for the general test of heterogeneity among the eGFR categories. Categorical variables are summarized by counts and percentages and compared using logistic regression analyses, with a general test of heterogeneity among the categories of eGFR, with and without adjustment for the confounding factors. To aid interpretation of the adjusted analyses, adjusted expected values were estimated for the continuous variables using the method of population adjusted means [17]. A corresponding approach was used for estimating expected proportions for the categorical variables. The relationships between baseline eGFR and each clinical outcome were assessed using Cox-proportional hazards models with e-GFR subdivided by the categories given above and treating the group with eGFR ≥ 60 ml/min/1.73 m^2^ as the referent. Evidence of a treatment by eGFR interaction was investigated with eGFR as a continuous variable to maximize statistical power. All analyses reported were adjusted for the following baseline confounders: country (Ireland, Netherlands, or Scotland); sex (male or female); smoking (current or not current); age; histories (yes/no) of each of hypertension, diabetes, and vascular diseases; low-density lipoprotein cholesterol (LDL-C); high-density lipoprotein cholesterol (HDL-C); systolic blood pressure (SBP); diastolic blood pressure (DBP); body mass index (BMI); fasting plasma glucose concentration. To explore the influence of inflammation on the results, the analyses were conducted with and without adjustment for C-reactive protein (CRP). Validity of the proportional hazards assumption was assessed by testing the significance of interaction terms between eGFR and the logarithm of time as a time dependent covariate.

## Results

Baseline eGFR data were available for 5,796 (99.9%) of the 5,804 randomised participants and this group forms the population whose data were analysed. The numbers (percentages) of participants in the prespecified categories of eGFR were 349 (6.0%), 1,104 (19.0%), 1,641 (28.3%), 2,702 (46.6%) in the categories (20–40), (40–50), (50–60), and ≥60 ml/min/1.73 m^2^, respectively.

Comparisons of baseline characteristics among the categories of eGFR are given in Table 1. In unadjusted analyses there are statistically significant associations between impaired renal function and older age, raised levels of low density lipoprotein cholesterol, body mass index, fasting plasma glucose and CRP, female sex and histories of hypertension, vascular disease and current smoking. In analyses adjusting for the skewing effect of study inclusion criteria most of the associations remain highly statistically significant and qualitatively unchanged with the exception of high density lipoprotein cholesterol (HDL-C), where there is now a strong statistical association with low levels of HDL-C, and for current smoking where, although the association is nominally statistically significant, the effect is attenuated.

**Table 1:**
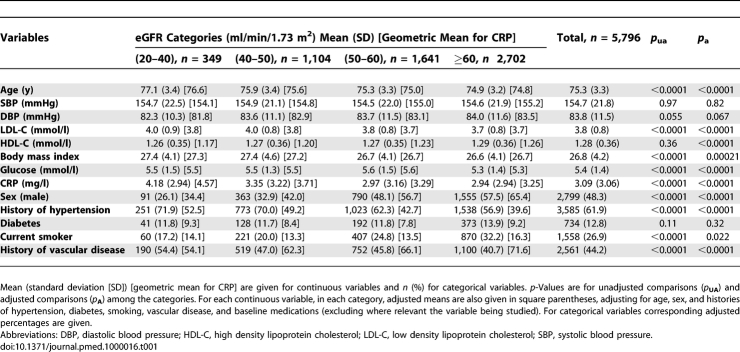
Baseline Characteristics Split by Baseline eGFR Category and Overall

Data on baseline cardiovascular medications are given in Table 2. There was no difference among the eGFR categories with respect to the use of ACE inhibitors, with or without adjustment for baseline confounders. For beta blockers, calcium channel blockers, nitrates, diuretics, and aspirin or anticoagulants there was evidence of higher use in the group with eGFR in the range (20–40) ml/min/1.73 m^2^. With the exception of diuretics, these differences were significantly attenuated after adjusting for baseline confounders. However, even in the adjusted analyses use of these medications was numerically highest in the range (20–40) ml/min/1.73 m^2^.

**Table 2:**
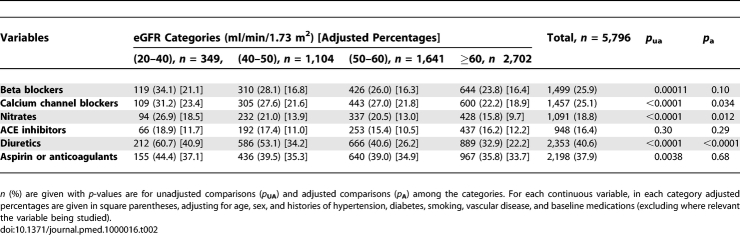
Common Baseline Cardiovascular Medications Split by Baseline eGFR Category and Overall

Table 3 contains details of event rates and estimated hazard ratios relative to the category ≥ 60 ml/min/1.73 m^2^ for the other categories of eGFR. When data were analysed without adjustment for levels of CRP, for all-cause mortality there was evidence of a greater than 2-fold increase in risk in the group with eGFR in the range (20–40) ml/min/1.73 m^2^ and a 40% increase in risk in the (40–50) ml/min/1.73 m^2^ group in comparison to the referent group. There was no association with cancer mortality. However, there was a strong association for vascular mortality mirroring the patterns seen for death from all causes. There was also a significant increase in risk of deaths from other causes (noncardiovascular, noncancer) in the (20–40) ml/min/1.73 m^2^ group. Although this category of 106 deaths contained a small number (*n =* 5) of deaths due to renal failure, the most common causes were pulmonary infection (*n =* 33), chronic obstructive pulmonary disease (*n =* 17), septicaemia (*n =* 6), and other infections (*n =* 5). There was evidence of substantial increases in risk in the (20–40) ml/min/1.73 m^2^ and (40–50) ml/min/1.73 m^2^ groups for coronary death or nonfatal myocardial infarction and for heart failure death or heart failure hospitalization. There was no significant association with the risk of stroke or transient ischaemic attack. There was at most a modest attenuation of the prognostic strength of eGFR after adjustment for levels of CRP (Table 3).

**Table 3:**
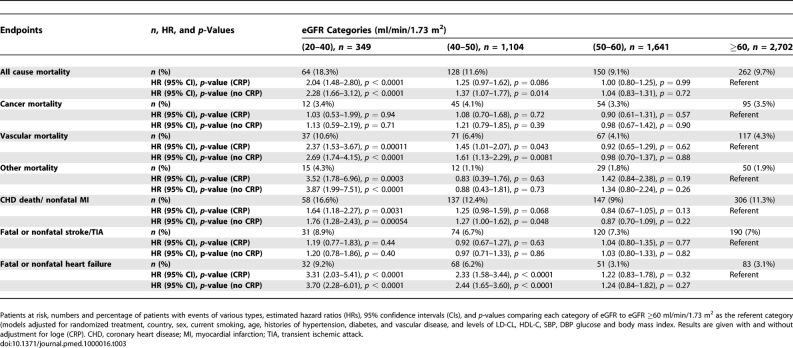
Patients and eGFR Categories

During follow-up, a total of 64 participants experienced serious adverse events classified as renal failure, 14 (4.0%), 20 (1.8%), 11 (0.7%), and 19 (0.7%) in the (20–40), (40–50), (50–60), and ≥ 60 ml/min/1.73 m^2^ groups respectively.

When tests for evidence of interaction between randomized treatment and eGFR in predicting each study outcome were carried out, the only result that achieved statistical significance was for the outcome of coronary heart disease death or nonfatal myocardial infarction (*p =* 0.021). In this case, there was a suggestion of greater benefit from pravastatin treatment relative to placebo in patients with a greater degree of impairment of eGFR, with a strong association between eGFR and outcome in the placebo group but not in the pravastatin group (Table 4).

**Table 4:**
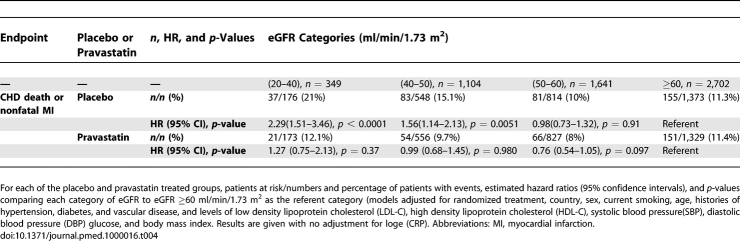
Treated Groups and eGFR Categories

There was no evidence of significant departures from the assumption of proportionality of hazards for any of the outcomes.

## Discussion

We have shown significant independent associations of reduced eGFR (below 50 ml/min/1.73 m^2^) with all-cause mortality, vascular deaths, coronary heart disease events (coronary death or nonfatal myocardial infarction), and for heart failure death or hospitalization, in an elderly population with vascular disease or vascular disease risk factors. There appeared to be a strong gradient of effect, with risks greatest in those with eGFR in the range (20–40) ml/min/1.73 m^2^, although significant increases in the incidence of these adverse events were also seen in the group with eGFR in the range (40–50) ml/min/1.73 m^2^. We found no evidence of increased risk associated with eGFR in the range (50–60) ml/min/1.73 m^2^ in comparison to the reference group (eGFR ≥ 60 ml/min/1.73 m^2^) for any of the outcomes studied. Reduced eGFR was not predictive of incident cerebrovascular events, or as expected, with cancer deaths. We have shown that presence of impaired renal function is independently associated with older age, obesity, raised fasting glucose levels, raised low-density lipoprotein cholesterol and reduced high-density lipoprotein cholesterol, female gender, history of vascular disease and hypertension. These relationships are generally what would be expected from the literature. We did not find any association with diabetes, with if anything slightly higher rates of diabetes in those with preserved eGFR. Lower muscle mass can in some elderly individuals lead eGFR to be higher than it otherwise would be. Since high hip circumference as a proxy for higher muscle mass is consistently shown to be protective for diabetes [18], then lower muscle mass would increase diabetes risk. This may be one possible explanation for our finding. Alternatively this could be an artefact of the inclusion/exclusion criteria of the study or a population selection effect in this elderly population, linked to the decreased prevalence of males and diabetics in those with eGFR < 60 ml/min/1.73 m^2^. The strong unadjusted association between smoking and high eGFR was surprising. However, this association is almost completely eliminated by adjustment for study-design confounders and is plausibly due to residual confounding.

Analyses of baseline cardiovascular medications revealed no evidence that participants with impaired renal function were less well treated. In middle-aged individuals with vascular disease the eGFR is predictive of all-cause, coronary heart disease and vascular mortality [1,2]. The predictive value in elderly patients has been uncertain. Our results contrast with those from lower-risk healthy community dwelling older individuals in whom eGFR appeared to have little predictive value [10,19]. Our data are from a high-risk elderly population, and in this context eGFR appears to have strong predictive value. In our high risk population with availability of carefully adjudicated nonfatal outcomes as well as fatal outcomes we were able to identify stronger associations at lower levels of eGFR than was possible in the analyses of data from the British Regional Heart Study [11]. In a study of even older patients from the Leiden 85-Plus Study [20] no association was found between baseline systolic blood pressure and impaired GFR. However, there was evidence in that study of statistically significantly lower eGFR levels at lower levels of diastolic blood pressure. In our study we found no association with systolic blood pressure and a nonstatistically significant trend suggesting slightly lower diastolic blood pressures in individuals with lower levels of eGFR. The lack of any statistically significant association between eGFR and cerebrovascular event risk is interesting. It is true that cerebrovascular events were less common than coronary events with lower associated power to detect any association. A large cross-sectional study of older adults has found an association of low GFR with prior stroke [21], however this has not been borne out in prospective cohorts. In the Rotterdam study Bos et al. [22], showed no significant association of low GFR with incident ischaemic stroke. Two other studies have also reported no association of low GFR with stroke [23,24]. It is noteworthy, in our study, that there was a lack of association between systolic blood pressure and eGFR.

Our results were not materially affected by adjustment for a measure of inflammation (CRP). There are a number of potential mechanisms by which low GFR may be associated with increased risk of adverse outcomes. Low GFR is associated with vascular risk factors including a history of hypertension and an unfavourable lipid profile, and an increased burden of underlying coronary atheroma [3]. This is likely to increase the risk of myocardial infarction and of death in those who have a coronary event. Hence, low eGFR could merely be a marker for cardiovascular risk rather than being causally implicated. However, in some situations low GFR may be a direct cause of vascular events or death. In heart failure, kidney disease is associated with impaired intra-cardiac conduction and progressive deterioration of diastolic function [6]. There are also associations with left-ventricular hypertrophy [7]. Further, inflammation, endothelial dysfunction, hypercoagulability, and raised homocysteine may play a role [11].

We identified a nominally statistically significant interaction between treatment with pravastatin and eGFR level for the outcome of coronary heart disease death or nonfatal myocardial infarction. The results are compatible with the increased risk associated with impaired renal function being eliminated in the group treated with pravastatin. We believe that this result should be treated cautiously because of the borderline level of significance, the fact that a number of tests of interaction were carried out, with one significant result, and because the result suggests a greater benefit of statin treatment in participants with the greatest degree of renal impairment, arguably the opposite to what might be expected. We also note that in the pooled analyses of earlier trials with pravastatin, admittedly in a younger population, such an interaction was not detected [25]. At the very least, these results suggest that patients with significantly impaired renal function benefit at least as much from statin treatment as do those with preserved renal function. Ongoing studies of the benefits of statin treatment in patients with chronic kidney disease are currently underway to investigate this patient population further [26].

There are a number of limitations to our study. We were restricted in our measures of renal function to eGFR. Cystatin-C may be a better predictor of outcomes, including in elderly patients [10,18]. We have no measure of albuminuria. The population that we studied was selected for a clinical trial with specific inclusion and exclusion criteria, and may not be fully representative of older people with vascular disease or vascular risk factors. There is the possibility that, despite careful adjustment, associations or the lack thereof between eGFR and baseline factors could be biased. Strengths of our study include assiduous follow-up and rigorous methods of classification of deaths and nonfatal clinical events. This high-risk population gave a large number of deaths and nonfatal vascular events, and therefore gives good statistical power to detect associations for a wide range of levels of eGFR.

In conclusion, we have established that impaired GFR, in an elderly population over the age of 70 y, is independently associated with significant levels of increased risk of all cause mortality and of fatal and nonfatal coronary and heart failure events. Our analyses of the benefits of statin treatment in relation to baseline renal function suggest that there is no reason to exclude elderly patients with impaired renal function from treatment with a statin.

## Abbreviations

CRP: C-reactive protein
DBP: diastolic blood pressure
GFR: glomerular filtration rate
HDL: high-density lipoprotein
LDL: low-density lipoprotein
SBP: systolic blood pressure
